# A Novel Occulta-Type Spina Bifida Mediated by Murine Double Heterozygotes *EphA2* and *EphA4* Receptor Tyrosine Kinases

**DOI:** 10.3389/fcell.2017.00105

**Published:** 2017-12-12

**Authors:** Nor Linda Abdullah, Siti W. Mohd-Zin, Azlina Ahmad-Annuar, Noraishah M. Abdul-Aziz

**Affiliations:** ^1^Faculty of Medicine, Department of Parasitology, University of Malaya, Kuala Lumpur, Malaysia; ^2^Faculty of Medicine, Department of Biomedical Science, University of Malaya, Kuala Lumpur, Malaysia

**Keywords:** *Epha2*^*tm1Jrui*^, *Epha4*^*rb-2J*^, neural tube, adhesion, fusion, spina bifida

## Abstract

Members of the Eph receptor tyrosine kinase have previously been implicated in cranial neural tube development. Failure of neural tube closure leads to the devastating conditions known as anencephaly and spina bifida. *EphA2* and *EphA4* are expressed at the tips of the closing spinal neural folds prior and during neural tube closure. We investigated the possible role of murine *EphA2* and *EphA4* during the last step of primary neural tube closure, which is adhesion and fusion. The individual mouse knockouts of *EphA2* and *EphA4 per se* do not exhibit neural tube defects (NTDs). The embryos generated by the crossing of double heterozygotes *Epha2*^*tm1Jrui/+*^*Epha4*^*rb-2J/+*^ displayed NTDs with a wide degree of severity including close exencephaly and close spina bifida (spina bifida occulta). Interestingly, mutants displaying NTDs had skin covering the underlying lesion. The tissue sections revealed the elevated neural folds had not adhered and fused. The phenotypes seen in *Epha2*^*tm1Jrui/+*^*Epha4*^*rb-2J/+*^ double heterozygous embryos suggest both genes play a compensatory role with each other in the adhesion and fusion of the neural tube. In this study, there exists a >50% penetrance of NTDs in the mouse mutants, which genetically have a single allele each of *EphA2* and *EphA4* absent.

## Introduction

The study of neural tube defects (NTDs) is regarded worldwide as a challenging field encompassing the understanding of embryology and the complications this common birth defect poses in the fields of neurosurgery and fetal surgery (Copp et al., 2015). Despite there being more than 20 years since the combined discovery of folic acid as a supplement to prevent NTDs and the landmark *in-utero* fetal repair of open spina bifida (Adzick et al., 1998), the rate of occurrence of spina bifida is still high and remains at 1 in a 1,000 births worldwide (Copp et al., 2015).

Neural tube closure is the product of successful primary neurulation that occurs in a developing embryo which gives rise to the central nervous system. Failure of primary neurulation is largely known to cause open spina bifida (spina bifida aperta) with neurological deficits among which myelochisis and myelomeningocele rank as the most severe phenotypes (Mohd-Zin et al., 2017). However, the complex mechanism of pathophysiology of close spina bifida with neurological deficits of which lipomyelomeningocele rank as the most severe phenotype have yet to be determined (May et al., 2013; Mohd-Zin et al., 2017).

Close spina bifida commonly known as spina bifida occulta have largely been shelved as a consequence of failure of secondary neurulation without having its mechanism properly elucidated (Copp et al., 2015; Mohd-Zin et al., 2017). Secondary neurulation occurs via cavitation of the mesenchymal rod and it is therefore absent of neural tissue. This would mean that secondary neurulation should not be present with neurological deficits apart from consequences of possible cord tethering (Adzick et al., 1998). According to Greene and Copp (2014), Copp et al. (2015) and Copp and Greene (2010), spina bifida occulta could only possibly occur due to perturbation of the secondary neural tube at the position of sacrum 2 and the subsequent sacral and coccyxgeal vertebrae (Copp and Greene, 2010; Greene and Copp, 2014; Copp et al., 2015). Our interest lies in the embryology of the occulta-type spina bifida with neurological deficits encompassing lipomyelomeningocele specifically in the lumbosacral region, that of which with higher level of lesion than sacrum 2 (May et al., 2013) and that of the embryology of brain malformations such as callosal dysgenesis with interhemispheric cyst (Edwards et al., 2014); all of which are characterized by a skin covering.

Studies have shown that the Eph receptor tyrosine kinases and their ephrin ligands are involved in embryonic development. In the early stages of embryonic development particularly during neural tube closure, *EphA2* and *EphA4* are expressed at the tips of the opposing neural folds in the spinal neural tube prior to adhesion and fusion during primary neurulation (Abdul-Aziz et al., 2009). The role of *EphA2* and *EphA4* in the developing neural tube have yet to be discovered, although it has been postulated that *EphA2* has a role during mammalian secondary neurulation (Naruse-Nakajima et al., 2001) and overexpression of *EphA4* in *Xenopus* could induce ectopic protrusion in the posterior end of the frog (Park et al., 2004).

The targeted mouse knockout of the *EphA2* gene (*Epha2*^*tm1Jrui/tm1Jrui*^) does not exhibit any gross anatomical defects (Ruiz and Robertson, 1994; Brantley-Sieders et al., 2004). The *Epha4*^*rb-2J/rb-2J*^ is a spontaneous mouse mutant that displays locomotor abnormalities of the hind limb resulting in a rabbit-like hopping movements and leaning phenotypes (Herrmann et al., 2010; Mohd-Zin et al., 2016). Considering the largely C57BL/6J background of both these strains which are publically available, we attempted to elucidate the potential compensatory roles of EphA2 and EphA4 seeing that the spatiotemporal pattern of expression of both these genes during neurulation is delineated at the tips of the opposing neural folds (Abdul-Aziz et al., 2009).

## Materials and methods

### Generation and genotyping of the *EphA2* and *EphA4* crosses

The B6;129S6-*Epha2*^*tm1Jrui*^/J strain (JAX EphA2 stock # 006028) and C57BL/6J-*Epha4*^*rb-2J*^/GrsrJ strain (JAX EphA4 stock # 003129) mutant mice were obtained from The Jackson Laboratory, Maine, United States. Genotyping of *Epha2*^*tm1Jrui/tm1Jrui*^ and *Epha4*^*rb-2J/rb-2J*^ mice was carried out according to the protocol provided by The Jackson Laboratory (stock # 006028 and stock # 003129 respectively) (Mohd-Zin et al., 2016). This study was carried out in accordance with the recommendations of Institutional Animal Care and Use Committee (IACUC) of University of Malaya. The protocol was approved by the Institutional Animal Care and Use Committee (IACUC)(# PAR/20/09/2011/NMAA).

### Embryo collection

*Epha2*^*tm1Jrui/tm1Jrui*^*Epha4*^+/+^ and *Epha2*^+/+^*Epha4*^*rb-2J/rb-2J*^ were intercrossed to generate a double heterozygous *Epha2*^*tm1Jrui/+*^*Epha4*^*rb-2J/+*^ line. The F2 generation of double heterozygotes *Epha2*^*tm1Jrui/+*^*Epha4*^*rb-2J/+*^ was timed-mated and embryos harvested at E11.5 (11.5 days post coitum). Pregnant females were euthanized by cervical dislocation and an incision was made at the abdominal area. The uterine horns were incised and immediately transferred into cold Dulbecco's Eagle Medium (DMEM) with 10% fetal bovine serum (FBS). The embryos were dissected out of the decidua and washed briefly with phosphate buffered saline (PBS) (Sigma) before overnight fixation in 4% paraformaldehyde (PFA) (Sigma). Subsequently, the embryos were washed and agitated in PBS for 10 min at 4°C. All steps from this point were agitated to ensure thorough washing. Then the embryos were dehydrated by ascending ethanol washes a concentration of 30, 50, and 70% for 20 min on each wash at 4°C. The embryos were kept in 70% ethanol at 4°C for downstream experiments. The embryos were analyzed in detail and documented under a high-resolution stereomicroscope (Leica MZ16).

### *In Situ* hybridization and RT-PCR

Whole-mount *in situ* hybridization, was performed using digoxygenin-labeled cRNA probes (Copp et al., 2000). Previously published probes were used for EphA2 and EphA4 (Flenniken et al., 1996; Gale et al., 1996).

### Scanning electron microscopy

Electron microscopy of embryos dissected out of deciduas was performed using the method and materials previously described (Abdul-Aziz et al., 2009). Imaging was then subsequently done on a JEOL FESEM (JSM-7001F) as previously described (Abdul-Aziz et al., 2009).

### Data analysis

The embryos collected were categorized both by phenotype and genotype. The data was presented as distribution according to genotype (Table 1) as well as incidence of phenotype according to genotype (Table 2).

**Table 1:** The genotypic distribution of the double heterozygotes *Epha2*^*tm*1*Jrui*/+^*Epha4*^*rb*−2*J*/+^ crosses in 5 litters harvested at E11.5.

	**Genotype**	***Epha4***		
		**+/+**	***rb−2J/+***	***rb−2J/rb−2J***
*Epha2*	+/+	2 (5%)	1 (2%)	1 (2%)
	*tm1Jrui/+*	1(2%)	34 (79%)	1 (2%)
	*tm1Jrui/ tm1Jrui*	1 (2%)	2 (5%)	N/A

**Table 2:** The phenotypic breakdown of the double heterozygotes *Epha2*^*tm1Jrui/+*^*Epha4*^*rb-2J/+*^ crosses in 5 litters harvested at E11.5.

**Phenotype**				
**SB**	**EX**	**Unturned with NTDs (Axial rotation defect)**	**Others (Cyclopia, caudal dysgenesis & gastrochisis)**	**Unaffected**
12 (28%)	7 (16%)	3 (7%)	3 (7%)	18 (42%)
*Epha2^*tm*1*Jrui*/+^Epha4^*rb*−2*J*/+^*	*Epha2^*tm*1*Jrui*/+^Epha4^*rb*−2*J*/+^*	*Epha2^*tm*1*Jrui*/+^Epha4^*rb*−2*J*/*rb*−2*J*^* & *Epha2^*tm*1*Jrui*/*tm*1*Jrui*^Epha4^*rb*−2*J*/+^*	*Epha2*^**tm*1*Jrui*/+*^*Epha4*^**rb*−2*J*/+*^	*Epha2^*tm*1*Jrui*/+^Epha4^*rb*−2*J*/+^, Epha2^+/+^Epha4*^+/+^, *Epha2^*tm*1*Jrui*/+^Epha4*^+/+^, *Epha2^+/+^Epha4*^+/rb−2J^, *Epha2^*tm*1*Jrui*/*tm*1*Jrui*^Epha4*^+/+^, & *Epha2^+/+^Epha4^*rb*−2*J*/*rb*−2*J*^*

*SB, Close Spina bifida (occulta), EX, Close exencephaly, NTDs, Neural Tube Defects*.

### Histology

Fixed embryos were embedded in paraffin wax, sectioned and stained with haematoxylin and eosin as previously described (Abdul-Aziz et al., 2009).

## Results

### *EphA2* and *EphA4* co-mediate neural tube adhesion and fusion during neural tube closure

**Figure 1:**
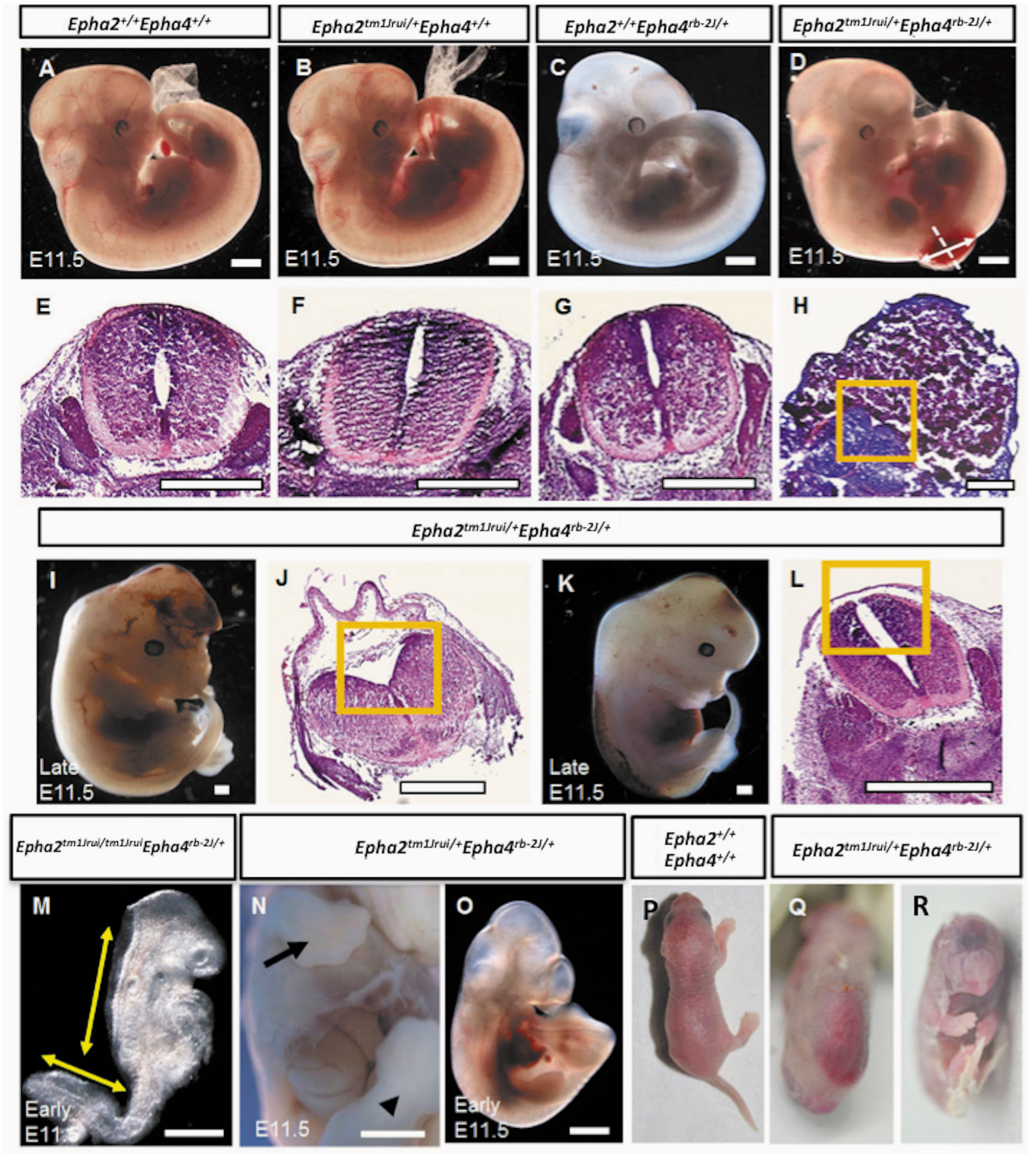
Phenotypes of the *Epha2*^*tm*1*Jrui*/+^*EphAa4*^*rb*−2*J*/+^ embryos and pups. **(A)**. Wildtype *Epha2*^+/+^*Epha4*^+/+^ embryo. **(B)** *Epha2*^*tm*1*Jrui*/+^*Epha4*^+/+^ embryo. **(C)** *Epha2*^+/+^*Epha4*^*rb*−2*J*/+^ embryo. **(D)**. Mutant E11.5 embryo with a close spinal neural tube defect (NTD). **(E–H)** The neural folds of the neural tube shown by transverse tissue sections of the embryos in **(A–D)** respectively. Dotted line in **(D)** indicates level of section in **(H)**. The diameter of the lesion is 0.75 mm (shown by double arrow). **(H)** Section of the lumbosacral sac revealing elevated neuroepithelium ensconced in cytoplasmic tissue. **(I)** Embryo with close exencephaly. **(J)** Cranial neural tube section revealed unfused cranial neural tube. **(K)** Embryo with close spinal neural tube. **(L)** Elevated neural folds failed to fuse. **(M)** An early E11.5 axial rotation defect embryo exhibiting elevated neural folds that failed to fuse from the cranial region down to the thoracic region and the unfused spinal neural folds. **(N,O)** Embryos with defects other than neural tube; **(N)** with gastrochisis and **(O)** with caudal dysgenesis. Forelimb and hindlimb in **(N)** embryo shown by arrow and arrow head respectively. **(P,Q)** A day old newborns. **(P)** Wildtype. **(Q)** Mutant pup with close NTD. **(R)** Mutant pup with cyclopia. Boxed region in **(H,J,L)** represents the opposing neural folds which failed to fuse. Scale bar represents 0.5 mm.

**Figure 2:**
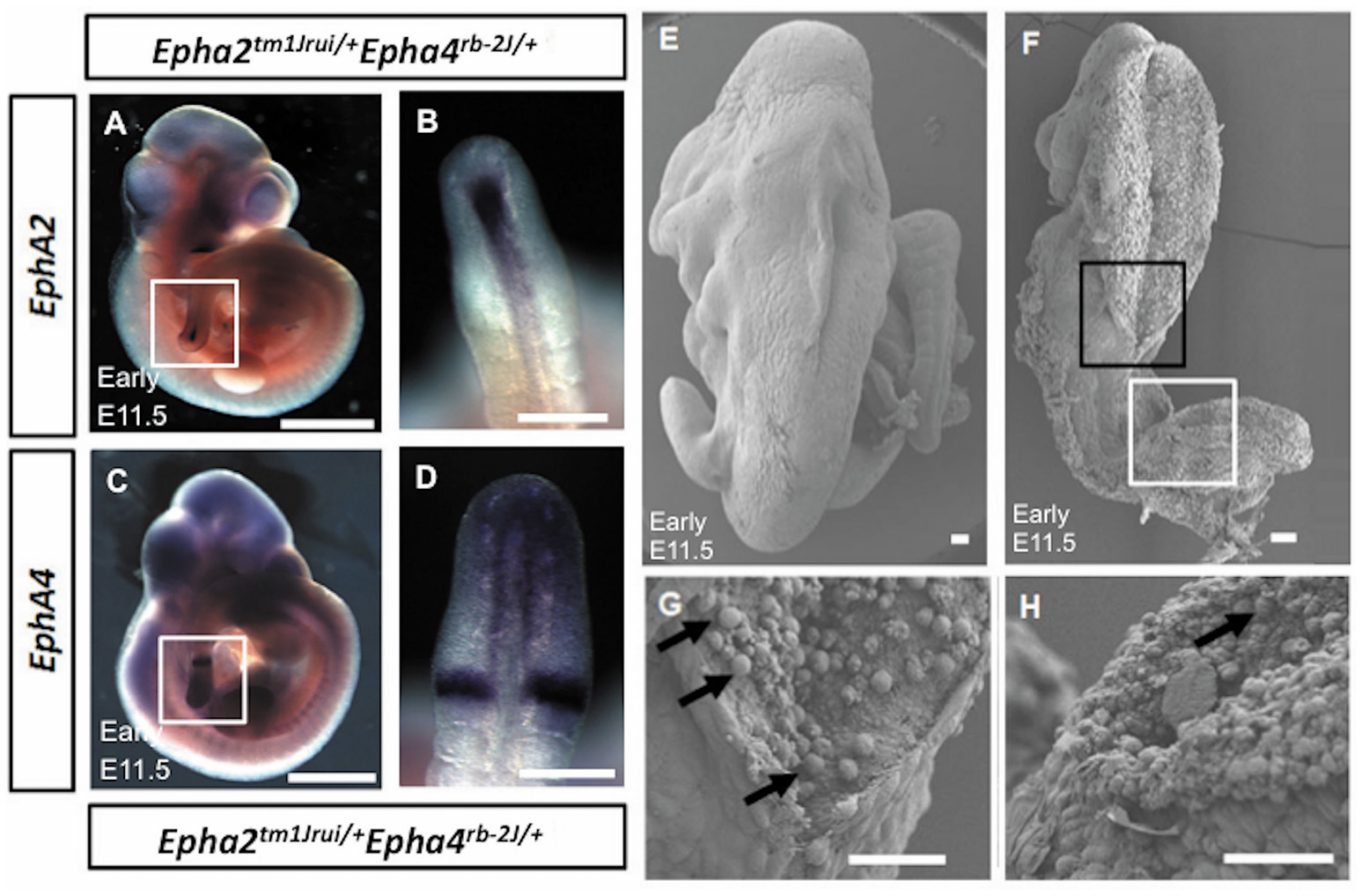
**(A–D)** Absence of expression of *EphA2* and *EphA4* at the point of adhesion and fusion in early E11.5 *Epha2*^*tm*1*Jrui*/+^*Epha4*^*rb*−2*J*/+^ embryos. The expression of *EphA2* **(A,B)** and *EphA4* **(C,D)** in the posterior neuropore of *Epha2*^*tm*1*Jrui*/+^*Epha4*^*rb*−2*J*/+^ but absence of *EphA2* and *EphA4* expression at the point of adhesion and fusion in the double heterozygotes respectively. White box in **(A,C)** represents the magnified version of the posterior neuropore as seen in B and D respectively. **(E–H)** Scanning electron micrograph of the axial rotation defect embryo (Figure 1M). **(E)** Scanning electron micrograph of a wild-type littermate of Figure 1M (E11.5). **(F)** The anterior (black box) and posterior (white box) neural tube of the early E11.5 embryo is open. **(G,H)**. Close ups of the closing point at the thoracic region **(G)** and opening site of the neural tube in the spinal region **(H)** respectively. The arrows in G represent potential apoptotic cells. Scale bar **(A–D)**: 1 mm; **(E–H)**: 0.05 mm

In this study, we have successfully generated a mouse neural tube defect model, which, mirrors the human spina bifida by using publically available mouse knockouts from JAX. By deleting the genes *EphA2* and *EphA4* at a specific location during neurulation, we observed a significant number of compound heterozygous embryos (*Epha2*^*tm1Jrui/+*^*Epha4*^*rb-2J/+*^) incapable of having a close neural tube that adheres and fuses in the region whereby these genes are expressed (Figures 1D,I,K). A wildtype littermate (*Epha2* ^+/+^*Epha4*^+/+^) of the E11.5 mouse embryo in Figure 1D does not exhibit any defect(s) as shown by Figures 1A,E. Haematoxylin and eosin staining of transverse section of the compound heterozygous embryos (*Epha2*^*tm1Jrui/+*^*Epha4*^*rb-2J/+*^) reveals bilateral elevated neural folds, which remain unfused in the dorsal midline at the point of closure of the neural tube highlighted by boxed region in the figure (Figures 1H,J,L). The unfused neural tube defect shown in Figure 1H was continuous at the site of lesion where the bump is located (shown in double arrows in Figure 1D). The diameter of lesion is 0.75 mm. Moreover, perturbation of both alleles of one gene and a single allele of the second gene simultaneously results in an unturned (*Epha2*^*tm1Jrui/+*^*Epha4*^*rb-2J/rb-2J*^ and *Epha2*^*tm1Jrui/tm1Jrui*^*Epha4*^*rb-2J/+*^) embryo (axial rotation defect) that successfully completes closure 1 (Figure 2E) as shown but display an open cranial and open spinal neural tube (Figure 1M) in 7% of the population of the EphA2 and EphA4 crosses (Table 2). The electron micrograph of embryo of Figure 1M, is visualized in Figures 2F,G,H. Cells appearing apoptotic were seen in electron micrographs of these embryos both in the anterior neuropore (Figure 2G) as well as the posterior neuropore (Figure 2H), which is the presumptive neural tube. This is unlikely to be caused by developmental delay as all 5 litters were harvested at E11.5. The wildtype phenotype ranges between early E11.5 to late E11.5.

### *EphA2* and *EphA4* play a role in the occulta-type neural tube defects

In this study we have shown that loss of an allele each of *EphA2* and *EphA4* simultaneously result in NTDs (close spina bifida and close exencephaly) in more than 50% of the population of double heterozygotes (*Epha2*^*tm1Jrui/+*^*Epha4*^*rb-2J/+*^). The embryos have lipomyelomeningocele (Figure 1D) and close cranial neural tube defect (Figure 1I) that is covered by the surface ectoderm, which is the presumptive skin. As many as 56% of our double heterozygotes exhibit a range of close NTD phenotype and can be seen with an unfused neural tube (Figures 1D,I,K) beneath a fully formed surface ectoderm (56% obtained from 34 double heterozygous embryos in a total of 5 litters). This is further confirmed by the phenotype seen also in a double heterozygote pup born with spina bifida occulta (close spina bifida) (Figure 1Q). The close spina bifida and close exencephaly phenotypes accounts for 44% of the total genotype of the 5 litters (Table 2).

Distribution of the phenotype in 5 litters obtained from the double heterozygotes *Epha2*^*tm1Jrui/+*^*Epha4*^*rb-2J/+*^ reveals a non-Mendelian inheritance of 5% (*Epha2* ^+/+^*Epha4*^+/+^): 79% (*Epha2*^*tm1Jrui/+*^*Epha4*^*rb-2J/+*^): 2% (*Epha2*^*tm1Jrui/+*^*Epha4*^*rb-2J/rb-2J*^): 5% (*Epha2*^*tm1Jrui/tm1Jrui*^*Epha4*^*rb-2J/+*^): unknown (*Epha2*^*tm1Jrui/tm1Jrui*^*Epha4*^*rb-2J/rb-2J*^) (Table 1). The phenotypic breakdown of these genotypes is as shown in Table 2. A small percentage of the double heterozygotes (7%) also exhibit caudal dysgenesis, gastrochisis and cyclopia (Figures 1N,O,R) apart from the neural tube defect phenotype encompassing close exencephaly and close spina bifida (44%). About 35% of the double heterozygotes have the appearance of normal, unaffected embryological development (Figures 1B,C,F,G). In contrast, the majority of double-knockout (*Epha2*^*tm*1*Jrui*/*tm*1*Jrui*^*Epha4*^*rb-*2*J*/*rb-*2*J*^) embryos were resorbed by E8.5. The parental double heterozygotes were considered robust without any gross abnormalities. Double heterozygous pups with abnormalities could not survive because the pups die within 2 h after birth (Figures 1P,Q) due to maternal neglect.

*EphA2* and *EphA4* gene expression was studied in the early E11.5 double heterozygotes (*Epha2*^*tm*1*Jrui*/+^*; Epha4*^*rb-*2*J*/+^) mutant embryos to observe for difference in expression pattern. Figure 2 (Figures 2A–D) showed the expression of the *EphA2* (Figure 2A) and *EphA4* (Figure 2C) in the posterior neuropore, but absence of expression at the point of adhesion and fusion (Figures 2B,D) in the double heterozygotes.

## Discussion

This finding demonstrates that the *EphA* genes play not only a compensatory role with each other, they also act synergistically among each other, the likeliest reason being the fact that this group of receptor tyrosine kinases which share similar characteristics as modulators of cell adhesion are able to rescue each other's functions (Hirai et al., 1987; Dravis et al., 2004). Evidence pertaining to this is widespread in many systems such as adhesion in the cloacal system being modulated by two Eph genes; adhesion of the palatal shelves are also modulated by two Eph genes as well as the formation of the corpus callosum which connects the left and the right side of the brain (Orioli et al., 1996; Dravis et al., 2004).

We have yet to genotype a double mutant (*Epha2*^*tm*1*Jrui*/*tm*1*Jrui*^*Epha4*^*rb-*2*J*/*rb-*2*J*^*)* among our crosses. However, we note that the numbers of animals given birth to in any of the crosses are much smaller in number than if the embryos were harvested during embryogenesis. There were between 10 and 12 embryos in each litter but if allowed to birth, the numbers dwindled to between 5 and 6 pups per litter. This gives rise to the possibility that if the embryos are unable to survive being a double heterozygote mutant, it gets resorbed; hence failure to complete embryogenesis successfully. Again, this system is similar to what has been observed in the EphB2; EphB3 double knockout (*EphB2*^−/−^ *EphB3*^−/−^*)* that suffer embryonic lethality (Orioli et al., 1996).

### Occulta-type neural tube defects mediated by *EphA2* and *EphA4* may act in a haploinsufficient manner

Our double heterozygotes (*Epha2*^*tm1Jrui/+*^*Epha4*^*rb-2J/+*^) have close spina bifida, which would translate clinically as spina bifida occulta. The implication of this finding is tremendous; that this is the first spina bifida occulta mouse model arising from failure of primary neurulation. Therefore, our mouse model can potentially explain the embryogenesis of lipomyelomeningocele as well as it is at odds with the current dogma of occulta-type spina bifida arising from failure of secondary neurulation. A previous study had reported *Trpm6*^*h*^ (Walder et al., 2009) to be spina bifida occulta with myelomeningocele. However, the gene expression information is lacking in the mutant *Trpm6*^*h*^ to understand the structure of the neural tube regulated by the *Trpm6*^*h*^ protein and whether this occurs during primary neurulation (Walder et al., 2009; Harris and Juriloff, 2010).

These mutant embryos showed gene dosage pattern whereby with every loss of an allele of *EphA2* and *EphA4* the phenotype representation would be more severe. A further 7% have a more severe neural tube defect when either both the EphA2 allele or both EphA4 allele is completely deleted simultaneously with a single allele of either EphA2 or EphA4 i.e., *Epha2*^*tm*1*Jrui*/+^*Epha4*^*rb-*2*J*/*rb-*2*J*^ or *Epha2*^*tm*1*Jrui*/*tm*1*Jrui*^*Epha4*^*rb-*2*J*/+^ (Figure 1M). Closure site 1 is never perturbed as shown in detail by the scanning electron micrograph (Figures 2F,G). This is most likely due to the spatiotemporal expression pattern of both *EphA2* and *EphA4* during neurulation (Figure 2H). *EphA2* and *EphA4* are not expressed at the closure 1 site during neurulation but are expressed in the rhombomeres and the posterior neuropore. This phenomenon also further illustrates the specificity of the perturbation of neural tube development in this model and supports the haploinsufficiency theory. Gene dosage determines severity of the phenotype. The defect is selective enough not to phenocopy craniorachischisis, yet its caudal and anterior neuropores remain remarkably open in the areas where *EphA2* and *EphA4* would be expressed in the wildtype. It is striking that Closure 1 is achieved (Shum and Copp, 1996). This would suggest that other Eph genes might be compensating the roles of *EphA2* and *EphA4* such as *EphA1* and *EphA5* which have a far broader expression domain than *EphA2* and *EphA4* (Abdul-Aziz et al., 2009). Further biochemical studies are required to understand the mechanism behind the interaction of *EphA2* and *EphA4* in neural tube closure.

### Multiple phenotypes exhibited by double heterozygotes

More than 65% of *Epha2*^*tm1Jrui/+*^*Epha4*^*rb-2J/+*^ shows a defective phenotype encompassing spina bifida occulta, close exencephaly, gastrochisis, caudal dysgenesis and cyclopia. It is interesting to note that in the double heterozygotes, the expression of the “dot” which is visible at the point of adhesion and fusion is absent (Figures 2B,D) in reference to Abdul-Aziz et al. (2009). The multiple phenotypes observed in *Epha2*^*tm1Jrui/+*^*Epha4*^*rb-2J/+*^ are most likely attributed to the *Epha4*^*rb-2J/rb-2J*^ mutant isoform that generates a protein size of 104 kDa (Mohd-Zin et al., 2016). Ephs and ephrins ability to exist in multiple forms capable of different functions for successful closure of the neural tube was demonstrated in Holmberg et al. (2000). Although *Epha2*^*tm1Jrui/tm1Jrui*^ is a complete targeted knockout of the EphA2 protein (Ruiz and Robertson, 1994; Brantley-Sieders et al., 2004), *Epha4*^*rb-2J/rb-2J*^ is not (Mohd-Zin et al., 2016). Furthermore, there are 17 mouse mutants with defective EphA4 protein further highlighting its complexity (Mohd-Zin et al., 2016). The variability in phenotypic representations due to differing mutations within the same *EphA4* gene were reviewed in Mohd-Zin et al. (2016). The fusion sites vary according to mouse strains (Detrait et al., 2005). Therefore, strains matter in the double heterozygotes *Epha2*^*tm1Jrui/+*^*Epha4*^*rb-2J/+*^. The background strain is a mix of C57BL/6J (Cook et al., 2004) and 129S6 (embryonic stem cell) (Brantley-Sieders et al., 2004). Double heterozygotes were not possible to be maintained on pure background, therefore viable and fertile double heterozygotes were used to generate mouse embryos and pups that were subsequently examined.

Skin covering of exencephaly; poses the question whether malformations of the brain such as callosal dysgenesis with interhemispheric cyst is implicated in neural tube closure as they occur during the period of neural tube closure or early during post-closure of the central nervous system (Barkovich et al., 2001; Edwards et al., 2014). Furthermore, expression of *EphA2* in the notochord could explain a potential role between Sonic hedgehog and the EphA receptor family seeing that the double heterozygotes in our study display cyclopia (Cooper et al., 1998; Abdul-Aziz et al., 2009). The observation of caudal dysgenesis among our double heterozygotes could also imply that the EphA receptor family is involved in both primary and secondary neurulation (Dravis et al., 2004; Weiss et al., 2014). This is not surprising as the most severe phenotype of the EphA2 knockout is a kinky tail that suggests its role in secondary neurulation (Naruse-Nakajima et al., 2001). Another interesting phenotype observed among our double heterozygotes is that of gastrochisis; *EphA2* is expressed in the region surrounding the gut, which could explain the potential role of the EphAs in gastrochisis.

## Conclusion

Our report provides the genotypic and phenotypic embryonic evidence of the occulta-type NTDs arising from failure of primary neurulation.

## Author contributions

NA, AA-A, and NA-A: Conceived and designed the experiments; NA, SM-Z, and NA-A: Performed the experiments; All authors: Analyzed the data; AA-A and NA-A: Contributed reagents, materials, analysis tools; NA and NA-A: Wrote the manuscript. All authors read and approved the manuscript for submission.

### Conflict of interest statement

The authors declare that the research was conducted in the absence of any commercial or financial relationships that could be construed as a potential conflict of interest.
